# The TT Genotype of the *KIAA1524 rs2278911* Polymorphism Is Associated with Poor Prognosis in Multiple Myeloma

**DOI:** 10.3390/cells12071029

**Published:** 2023-03-28

**Authors:** Aneta Szudy-Szczyrek, Radosław Mlak, Marcin Mazurek, Tomasz Krajka, Sylwia Chocholska, Paulina Bitkowska, Marta Jutrzenka, Michał Szczyrek, Iwona Homa-Mlak, Andrzej Krajka, Teresa Małecka-Massalska, Marek Hus

**Affiliations:** 1 Department of Haematooncology and Bone Marrow Transplantation, Medical University of Lublin, 20-059 Lublin, Poland; 2 Body Composition Research Laboratory, Department of Preclinical Sciences, Medical University of Lublin, 20-059 Lublin, Poland; 3 Department of Human Physiology, Medical University of Lublin, 20-059 Lublin, Poland; 4 Division of Mathematics, Department of Production Computerisation and Robotisation, Lublin University of Technology, 20-618 Lublin, Poland; 5 Collegium Medicum, University of Warmia and Mazury in Olsztyn, 10-719 Olsztyn, Poland; 6 Department of Pneumonology, Oncology and Allergology, Medical University of Lublin, 20-059 Lublin, Poland; 7 Institute of Computer Science, Maria Curie-Sklodowska University, 20-033 Lublin, Poland

**Keywords:** CIP2A, MYC, multiple myeloma, polymorphism, prognosis, molecular biomarkers

## Abstract

Background: The *KIAA1524* gene encodes an oncoprotein, CIP2A, which inhibits the phosphorylation of the Akt kinase B, stabilizes the c-Myc protein, and, through that, promotes cancerogenesis. An increase in CIP2A expression has been observed in numerous solid tumors and hematologic malignancies, including multiple myeloma (MM). The aim of our study was to evaluate the clinical impact of the functional single nucleotide polymorphisms (SNP) of the *KIAA1524* gene (rs2278911, 686C > T) in MM patients. Methods: The study group consisted of 128 patients with de novo MM. EDTA venous blood samples were collected prior to the treatment. The SNPs were analyzed by Real-Time PCR with the use of specific Taqman probes. Results: Multivariable analysis revealed that variables independently associated with shorter progression-free survival (PFS) included thrombocytopenia, *delTP53* and *IGH/CCND1* translocation and the TT genotype of the *KIAA1524* gene (686C > T) (median PFS: 6 vs. 25 months; HR = 7.18). On the other hand, autologous haematopoietic stem cell transplantation (AHSCT) was related to a lower risk of early disease progression. Moreover, light chain disease, International Staging System (ISS) 3, poor performance status, hypoalbuminemia, *IGH/FGFR3* translocation and the TT genotype of the *KIAA1524* gene (686C > T) were independent prognostic factors associated with shorter overall survival (OS) (median OS: 8 vs. 45 months; HR = 7.08). Conclusion: The evaluation of the SNP 686C > T of the *KIAA1524* gene could be used as a diagnostic tool in MM patients at risk of early disease progression and death.

## 1. Introduction

Multiple myeloma (MM) is a malignant neoplasm characterized by the clonal proliferation of atypical plasma cells within the bone marrow, and accounts for around 10% of all hematologic malignancies [1]. The risk factors for MM include age, race, sex, family history, chronic inflammation, immunodeficiency and exposure to radiation and organic solvents [2,3,4,5]. Patients may experience a wide range of unspecific and usually mild symptoms, including fatigue, nausea, vomiting, weight loss and recurring infections [3]. At the time of presentation, 73% of the patients have anemia, almost 80% have osteolytic bone disease and 19% have acute kidney injury [6]. The imbalance between the osteoblasts’ and the osteoclasts’ activity results in the development of bone lesions [7]. The progression of the disease leads to bone fractures, osteoporosis, multi-organ failure and death [3].

The current mainstay of MM treatment includes proteasome inhibitors (PI) and immunomodulatory drugs (IMiDs) known as monoclonal antibodies. Autologous hematopoietic stem cell transplantation (AHSCT) remains the standard of care for young patients with newly diagnosed MM [7,8]. Although the introduction of new drugs has significantly improved the treatment’s results, MM remains a disease with a very unpredictable course, with the estimated survival ranging from less than six months to more than 10 years [9]. The clinical outcome of MM is known to be affected by several factors, including the patient’s performance status, age, comorbidities, therapy choice, microenvironment, tumor burden and characteristics of the tumor—including the chromosomal changes and gene expression. However, even within the same risk groups, we observe a significant heterogeneity of the outcome [4,10,11].

The MM cells derive from B-line cells, which develop in the germinal centers located within the lymph nodes and the spleen. Malignant development begins with the acquisition of hyperdiploidy or translocation with the immunoglobulin heavy chain (*IGH*) gene locus [4]. Based on the chromosome ploidy and other parameters, genetic abnormalities are divided into the hyperdiploidy karyotype, which is usually associated with translocations of *IGH* locus, and the hypodiploid karyotype, which is mostly linked to trisomies [12]. The first stage of the malignant transformation is the B cell’s immortalization. One of the most common initiating mutations, observed in nearly 50% of MM patients, is a chromosomal translocation with the *IGH* locus at the 14q32 region, which may involve many other loci, such as 11q13, 4p16.3, 6p21, 16q23 or 20q11 [12,13]. Hyperdiploidy is present in 55% of MM patients and is associated with trisomies of chromosomes such as 3, 5, 7, 9, 11, 15, 19 and 21 [12,14]. While trisomies 3 and 5 are beneficial for patients’ survival, trisomy 21 is linked with poor prognosis [14]. The genomic instability promotes further molecular changes. The most common somatic mutations, which are present in around 50% of patients, occur in genes of the *RAS/MAPK* pathway, such as *KRAS*, *NRAS* and *BRAF* [4]. Other cytogenic changes have been described as secondary genetic aberrations, among which the most frequent are *MYC* rearrangements, del (13q), del (17p), (del)1p and amp(1q) [10,12]. These have been connected to MM progression.

The signal pathways JAK2/STAT3, Ras/Raf, PI3K/AKT and NFκB have been identified as those in control of MM cells’ apoptosis, migration and proliferation, and may be linked to MM cells’ drug resistance [15,16]. The interactions between MM cells and non-malignant cells from the bone marrow microenvironment—such as stromal and endothelial cells—contribute to elevated levels of interleukin 6 (Il-6), vascular endothelial growth factor (VEGF) and insulin-like growth factor 1 (IGF-1), which promote the PI3K/Akt/mTOR pathway’s activation [17]. As that signaling pathway regulates a wide range of MM’s cellular processes, it is under intense research for targeting treatment [16,17]. So far, its role in the development of MMs has not been fully explained; however, in most other neoplasms, its activity is controlled by phosphatases such as PP2A (protein phosphatase 2).

The *KIAA1524* gene encodes the cancerous inhibitor of PP2A (CIP2A)—also known as a p90 tumor-associated antigen—which is a human oncoprotein whose overexpression has been observed in patients with solid tumors of the head and neck, breast, stomach and large intestine [18]. The c-Myc stabilization in the tumor cells is promoted by the CIP2A thanks to its ability to inhibit the c-Myc protein’s dephosphorylation, which is mediated by the activity of PP2A towards c-Myc serine 62 [18,19]. Additionally, CIP2A is also responsible for the downregulation of protein kinase B (Akt 9s) phosphorylation [20]. Myc and CIP2A appear to promote each other’s expression in a positive regulatory loop [21]. As the inhibition of CIP2A may allow for the PP2A-mediated proteolytic degradation of c-Myc in cancer cells and thereby limit the cancer cells’ growth and transformation, the CIP2A may be a promising candidate for targeted treatment [18,19].

## 2. Materials and Methods

### 2.1. The Study Population

The study group included 128 (64 male and 64 female) newly diagnosed MM patients who received first-line chemotherapy with thalidomide (CTD—cyclophosphamide + thalidomide + dexamethasone; *n* = 27; 21.1%), bortezomib (V(C)D—bortezomib + cyclophosphamide + dexamethasone; *n* = 58; 45.3%) or bortezomib and thalidomide (VTD—bortezomib + thalidomide + dexamethasone; *n* = 43; 33.6%). Patients were recruited in the Department of Hemato-oncology and Bone Marrow Transplantation in Lublin from 2015 to 2020.

MM diagnosis was based on SLiM CRAB criteria, according to the International Myeloma Working Group (IMWG) recommendations. Disease staging was determined according to Durie–Salmon and the current ISS staging system. Treatment response was assessed according to IMWG 2016 consensus criteria [22,23,24,25]. The severity of adverse effects was evaluated using CTCAE (version 5.0) [26].

Detailed patients’ characteristics are presented in Table 1.

### 2.2. DNA Isolation

The DNA isolation from 200 μL of whole peripheral blood was performed using the column method with a dedicated kit, according to the manufacturer’s recommendations (DNA Blood Mini Kit, Qiagen, Toronto, ON, Canada). Subsequently, the spectrophotometric evaluation of the concentration and quality of the obtained DNA was carried out using a NanoDrop Lite Spectrophotometer (Thermo Fisher Scientific, Waltham, MA, USA).

### 2.3. Single Nucleotide Polymorphism Analysis

A StepOnePlus Real-Time PCR System (Applied Biosystems, Foster City, CA, USA) was used for the SNP genotyping. The real-time PCR was performed according to the manufacturer-established protocol with the use of the Genotyping Master Mix and TaqMan probes specific for the *KIAA1524* SNP (rs2278911; 686C > T) (C__11237075_30, Thermo Fisher Scientific, USA). A selected probe was functionally tested by the manufacturer. The qPCR was performed in 10 µL reaction volume on 96-well plates. A rection mix containing 5 µL of Taqman Genotyping Master Mix, 0.5 µL of Taqman SNP assay (20×) and 4.5 µL of 0.2 ng/µL diluted DNA template was added to each well. The thermal cycling protocol was as follows: enzyme activation at 95 °C, 40 cycles of denaturation at 95 °C for 15 s and annealing at 60 °C for 1 min. All sample tests were performed in triplicate. After the amplification, the obtained genotypes were analyzed with StepOne Software v2.3 (Applied Biosystems, Waltham, MA, USA). Additionally, 10% of the samples were randomly selected and re-analyzed in a sequencing device (3500 Genetic Analyzer, Life Technologies). A 100% consistency of the results was obtained.

### 2.4. Plasma Cells Isolation

The bone marrow mononuclear cells (BMMC) were isolated from 3 mL of bone marrow (diluted 10×) using the density gradient centrifugation method (Biocoll, AG Biochrom, Berlin Germany), then subjected to washing and a viability assessment followed by the cell separation. The cell separation was performed after the incubation with CD138 antibodies and conducted according to the manufacturer’s instructions with the use of the magnetic-activated cell sorting technique (MACS^®^ Cell Separation, Bergisch Gladback, Germany). Subsequently, the assessment of viability and quantity of plasma cells was performed.

### 2.5. Gene Expression in the Bone Marrow Plasma Cells

Total RNA was isolated from the plasma cells (the maximum of 10^7^ cells were used) with the use of an RNeasy Mini Kit (Qiagen, Toronto, ON, Canada). The expression of the *KIAA* and *MYC* genes was measured with the use of a StepOnePlus device and a dedicated High-Capacity cDNA Reverse Transcription Kit, TaqMan™ Fast Advanced Master Mix and TaqMan probes (Thermo Fisher Scientific, USA). For the *KIAA1524* (assay ID: Hs00405413) and *MYC* (assay ID: hs00153408) genes, the *ACTB* (assay ID: hs99999903) gene was used as the reference gene. The relative expression of the investigated molecular targets was calculated using the 2^−Δ*C*t^ and 2^−ΔΔ*C*t^ formulas.

### 2.6. Cytogenetic Assessment

The bone marrow samples were subjected to cytogenetic analysis. Abnormalities, such as the *TP53* gene deletion, *IGH* gene rearrangements and *CKS1B* gene amplification, were detected by simultaneous staining of cytoplasmic immunoglobulin with the fluorescence in situ hybridization (cIg-FISH) technique according to the recommendations of Ross et al., with some modifications [27]. The following probes, all from Abbott Molecular (Abbott Park, IL, USA), were used: Vysis TP53/CEP 17 FISH Probe Kit for detection of del(17p13.1), Vysis IGH/FGFR3 Dual Colour, Dual Fusion Translocation Probe for detection of t(4;14)(p16;q32), Vysis IGH/MYC/CEP 8 Tri-colur, Dual Fusion Translocation Probe for detection of t(8;14)(q24;q32), Vysis IGH/CCND1 Dual Colour, Dual Fusion Translocation Probe for detection of t(11;14)(q13;q32), Vysis IGH/MAF Dual Colour, Dual Fusion Translocation Probe for detection of t(14;16)(q32;q23) and Vysis 1q21 CKS1B SpectrumOrange/1p32 CDKN2C SpectrumGreen FISH Probe Kit for the detection of amp(1q32). Fluorescent microscopic analysis was performed by scoring 100 AMCA-positive (anti-mitochondrial antibody-positive) plasma cells to determine the frequency of each aberration. The cut-off values were set at 20% for deletion/amplification probes and 10% for dual fusion probes, according to the recommendations of the European Myeloma Network [15,16].

### 2.7. Statistical Analysis

MedCalc software (v.15.8) was used for statistical analysis of the acquired data. A c*hi-*square test was performed to assess the *KIAA1524* genotypes’ distribution depending on the selected demographic, as well as clinical and molecular variables. Due to the abnormal distribution of the studied continuous data in the D’Agostino–Pearson test, either the non-parametric Mann–Whitney U Test (for comparing two independent groups) or the ANOVA Kruskal–Wallis test (for comparing more than two independent groups) was performed in order to compare the *KIAA1524′s* expression levels depending on the distribution of SNP located in this gene. The Spearman’s rank correlation coefficient test was applied to assess the correlation between the expression of the *KIAA1524* gene and the *MYC* gene.

The influence of selected demographic, clinical and molecular variables on the risk of no response to chemotherapy was estimated by the odds ratio (OR) and corresponding 95% confidence intervals (CI). In terms of the survival evaluation, the log-rank test was applied for the univariable analysis, whereas Cox regression models were used in the multivariable analysis. The influence of selected demographic, clinical and molecular variables on the risk of death was described by the hazard ratio (HR) and corresponding 95% CI. The Kaplan–Meier estimation method was utilized to generate the survival curves, whereas forest plot graphs were used to illustrate the results of the multivariable analysis. In cases in which both the composite (e.g., disease stage according to ISS) and its constituent variables (e.g., albumin or B2M levels) were statistically significant according to the univariable analysis, only the first one of them was included in the multivariate models. The backward elimination method was used for the selection of variables to be included in the results adjustment. Finally, in the multivariable PFS analysis, all results were adjusted by the following variables: gender, AHSCT, PLT, *del17/TP53*, *t(11;14) IGH/CCND1* and *KIAA* gene genotype. On the other hand, in the multivariable OS analysis, all results were adjusted by diagnosis, performance status, anemia before treatment, *t(4;14) IGH/FGFR3* and *KIAA* gene genotype. The progression-free survival (PFS) was defined as the period between the start of the treatment and the occurrence of disease progression (complete data), or of the last documented follow-up (censored data). The overall survival (OS) was calculated from the start of treatment until the patient’s death (complete data) or the last documented follow-up (censored).

The data from the MMRF CoMMpass project were retrieved from “https://portal.gdc.cancer.gov/ (accessed on 21 March 2023)” through the R language wrapper library GenomicDataCommons (installed by BiocManager). Based on the obtained data, Pearson correlations between genes of interest were computed, and further selected results were illustrated with the use of the corrplot library (with heat maps presenting correlation matrix).

In all analyses, results with a *p*-value < 0.05 was considered statistically significant. Additionally, *p*-values in the range of 0.05–0.06 were considered to show a trend toward statistical significance.

## 3. Results

### 3.1. The KIAA1524 Genotypes’ Distribution in Relation to the Demographic, Clinical and Molecular Factors

The only significant difference observed between the patients with various *KIAA1524* genotypes was the prevalence of the *t(4;14) IGH/FGFR3*, whose absence was more common in patients with the CC genotype than in the carriers of the T allele (80% vs. 20%, *p* = 0.0095). The detailed data is presented in the Supplementary Table S1.

### 3.2. Response to Treatment

A significantly higher risk of non-response after the 2nd cycle of chemotherapy was observed in patients with poor performance status (PS: 2–4: OR = 3.95), those who were treated with a CTD regimen (OR = 5.35), those with elevated CRP (OR = 3.32) and those with the TT genotype of the KIAA1524 gene (OR = 8.33). On the other hand, the risk of non-response after the 2nd cycle of chemotherapy was significantly lower in patients treated with the VTD regimen (OR = 0.05) and those with the CC genotype of the *KIAA1524* gene (OR = 0.23). In turn, a significantly higher risk of non-response after the 4th cycle of chemotherapy was observed in patients with elevated CRP (OR = 6.70), those with the presence of *t(11.14)/CCND1* translocation (a trend toward significance; OR = 6.42) and those who were carriers of the TT genotype of the KIAA1524 gene (OR = 20.20). On the other hand, a significantly higher risk of non-response after the 6th cycle of therapy was observed in patients with thrombocytopenia (OR = 9.22), those with the presence of 17p/TP53 deletion (OR = 12.46) and those with the presence of *t(11.14)/CCND1* translocation (OR = 55.20). Moreover, a significantly higher risk of non-response after the 8th cycle of therapy was observed in patients with the presence of *t(11.14)/CCND1* translocation (OR = 16.00). Detailed data on the influence of the selected demographic, clinical and molecular variables on chemotherapy response are presented in Supplementary Table S2.

### 3.3. Progression-Free Survival

#### 3.3.1. Univariable Analysis

**Figure 1:**
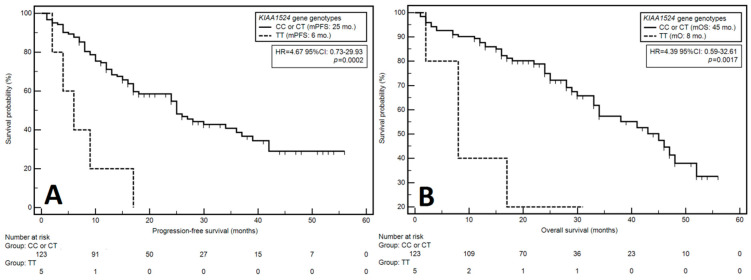
Kaplan–Meier curves presenting the influence of KIAA gene genotypes on progression-free survival (**A**) and overall survival (**B**).

According to the univariable analysis, the factors significantly correlated with an increased risk of PFS reduction were: male sex (HR = 1.65), stage 3 disease according to the ISS classification (HR = 1.74), higher stage of chronic kidney disease (>G2: HR = 1.85), poor performance status (PS: 2–4; HR = 1.60), anemia before treatment (HR = 2.40) low platelet count (HR = 2.08), low albumin level (HR = 2.29), high LDH level (HR = 1.95), high β2-microglobulin level (B2M) (HR = 2.58), high level of creatinine (HR = 1.64), the presence of *del17p/TP53* (HR = 1.84) and the presence of *t(11;14) IGH/CCND1* (HR = 2.49). Additionally, a higher risk of PFS reduction was observed in patients with the TT variant of the *KIAA1524* gene (rs2278911) as compared to patients with other variants (median PFS: 6 vs. 25 months; HR = 4.67) (Figure 1A). A decreased risk of PFS reduction was observed in patients treated with VTD as compared to those treated with CTD or V(C)D schemes (HR = 0.56), those in whom AHSCT was applied (HR = 0.37) and those with the CC genotype (compared to carriers of the T allele, HR = 0.40).

#### 3.3.2. Multivariable Analysis

**Figure 2:**
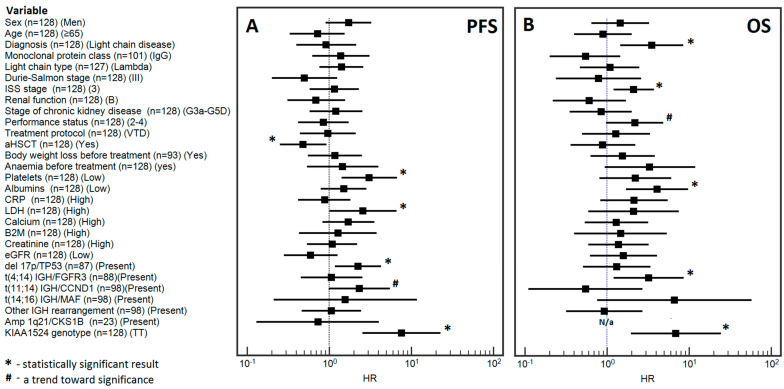
Forest plot showing the results of multivariate analysis for progression-free survival (**A**) and overall survival (**B**).

Multivariable analysis confirmed that thrombocytopenia (HR = 3.07), high LDH level (HR = 2.59), presence of *del17p/TP53* (HR = 2.25), presence of t(*11;14) IGH/CCND1* (a trend toward significance; HR = 2.34) and the TT genotype of the *KIAA1524* gene (rs2278911) (HR = 7.57) were independent prognostic factors for a higher risk of progression. However, AHSCT was the only independent prognostic factor for a lower risk of progression (HR = 0.48) [Figure 2A].

### 3.4. Overall Survival

#### 3.4.1. Univariable Analysis

The univariable analysis identified the following factors as significantly related to higher risk of OS death: light chain disease (HR = 2.37), stage 3 disease according to the ISS classification (stage 3; HR = 2.03), higher stage of chronic kidney disease (>G2: HR = 1.78), poor performance status (PS: 2–4; HR = 2.01), the presence of anemia before treatment (HR = 2.43), low albumin levels (HR = 2.28), high B2M levels (HR = 2.73), high creatinine levels (HR = 1.74), the presence of *del17p/TP53* (HR = 1.84) and the presence of *t(11;14) IGH/CCND1* (HR = 2.62). Moreover, we observed a higher risk of death in patients with the TT variant of the *KIAA1524* gene *(rs2278911)* as compared to those with other genotypes (median OS: 8 months vs. 45 months; HR = 4.39) (Figure 1B). A significantly lower risk of OS reduction was observed in the subjects in whom AHSCT was applied (HR = 0.53) and those with the CC variant of the *KIAA1524* gene (*rs2278911*) as compared to carriers of the T allele (HR = 0.41).

#### 3.4.2. Multivariable Analysis

The multivariable analysis confirmed the independent prognostic value of the following factors: light chain disease (HR = 3.52), the stage 3 disease according to the ISS classification (HR = 2.11), performance status (a trend toward significance; HR = 2.19), low albumin levels (HR = 4.08), the presence of *t(4;14)*, *IGH/FGFR3* (HR = 3.22) and the presence of the TT genotype of the *KIAA1524* gene *(rs2278911)* (HR = 6.89) [Figure 2B]. 

A graphical representation of the results of the multivariable analysis of PFS and OS is shown in Figure 2. Detailed data on the influence of the selected demographic, clinical and molecular variables on PFS and OS are presented in Supplementary Table S3.

### 3.5. Relationship between the Expression of the KIAA1524 Gene, Its SNP (rs2278811) and the Expression of the MYC Gene

**Figure 3:**
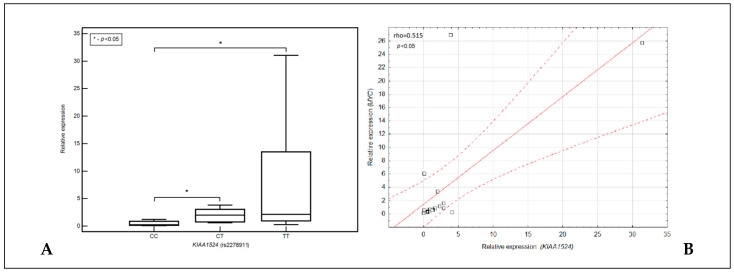
Box–whisker graph presenting a comparison of expression of the *KIAA1524* gene depending on genotypes of the SNP of this gene (**A**). Distribution plot presenting correlation between expression of *KIAA1524* gene expression of the *MYC* gene (**B**).

We noted that the expression of the *KIAA1524* gene was significantly lower in the carriers of the CC genotype of this gene compared to the carriers of the CT and TT genotypes (in both cases *p* < 0.05) (Figure 3A). Moreover, we found an intermediate, positive correlation between expression of the *KIAA1524* gene and the *MYC* gene (rho = 0.515; *p* = 0.0345) (Figure 3B).

### 3.6. Relationship between the Expression of the KIAA1524 Gene and the Expression of Other Genes Based on Data from the CoMMpass Project

**Figure 4:**
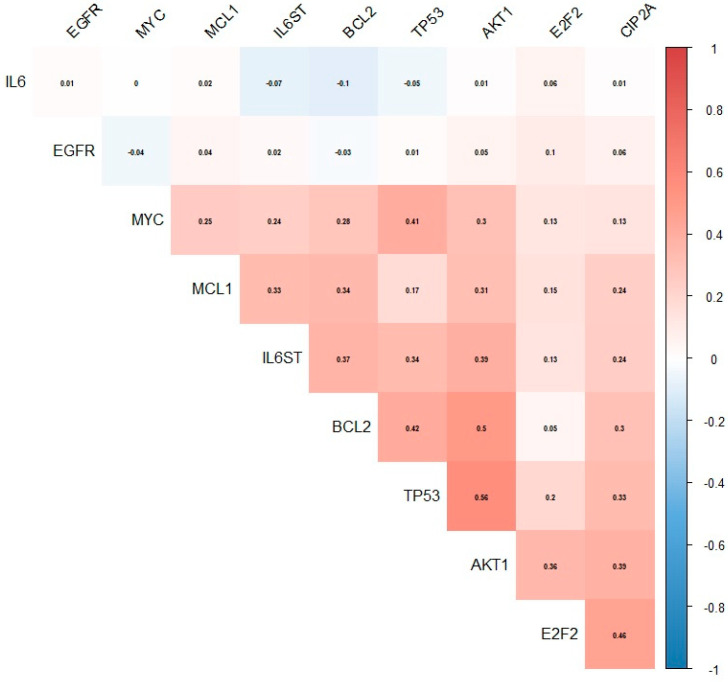
Heat map presenting correlation matrix, including *CIP2A/KIAA1524* and selected genes involved in the biology or pathomechanism of MM, created based on data retrieved from the CoMMpass project.

**Figure 5:**
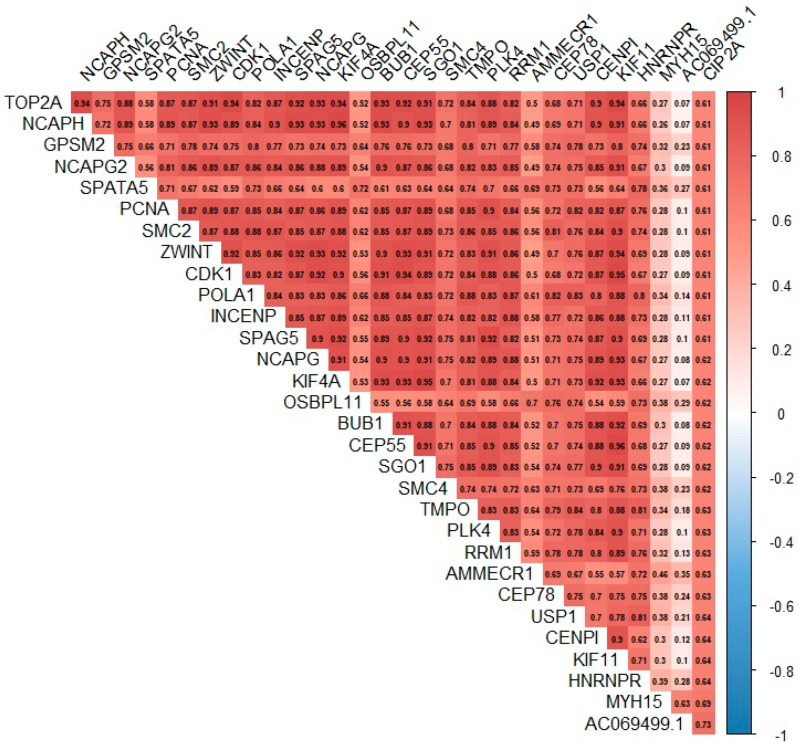
Heat map presenting correlation matrix including 30 genes demonstrating the strongest correlation with *CIP2A/KIAA1524,* created based on data retrieved from the CoMMpass project.

Based on data from the CoMMpass project, we assessed the correlation between the expression of the *KIAA1524* gene (known in the above database as *CIP2A*) and other genes known to be involved in the biology and pathomechanism of MM (Figure 4). A statistically significant and positive correlation between the expression of the *CIP2A* gene and the *TP53*, *IL6ST*, *MCL1*, *BCL-2*, *AKT1* and *E2F1* genes was found. In addition, a positive correlation between the expression of *CIP2A* and the *MYC* gene, which was demonstrated in our study, was further validated in the data retrieved from this project. Moreover, we found that the studied gene was strongly correlated with genes involved, among others, in the regulation of the cell cycle and differentiation, DNA replication, DNA damage detection and repair, mRNA processing and transport, spindle dynamics, chromosome processing and segregation (Figure 5).

## 4. Discussion

MYC affects a number of metabolic pathways and plays a crucial key role in the development of MM. It plays a role in cycle regulation, proliferation, differentiation and metabolism of the cells, and its dysregulation has been shown to promote the transition from monoclonal gammopathy of undetermined significance (MGUS) to MM. Its activity can be regulated by its mutations, as well as by changes in pathways involved in its regulation (e.g., it may be controlled by mTORC1 and RAS on a post-transcriptional level) [28,29,30].

The two most important phosphorylation sites on MYC, responsible for its stabilization and degradation are Serine62 (S62), which stabilizes MYC and participates in the target gene selection when phosphorylated, and Threonine 58 (T58), which runs the MYC degradation when phosphorylated [30,31]. S62 is phosphorylated by the extracellular-regulated kinases (ERKs) 1 and 2, while the glycogen synthase kinase (GSK-3β) is responsible for the T58′s phosphorylation, which can only be carried out after the S62′s phosphorylation [30]. Before MYC can be delivered to and degraded by the ubiquitin–proteasome system, it must be dephosphorylated in position S62 by PP2A [32,33]. CIP2A inhibits the PP2A, and, through that, stabilizes MYC [30,32]. Moreover, PP2A’s inhibition increases ERK’s activation and promotes cell proliferation and survival, as well as the expression of invasive proteases [30].

Since CIP2A also influences the RAS pathway, it may indirectly regulate the MYC pathway through its elements [34,35,36] The Ras proto-oncogene has been shown to enhance the ERK’s activity, restrict the GSK-3β’s activity and affect the PI3K pathway-mediated T58′s phosphorylation, making it a MYC stabilizer [30]. In addition, the mutations in MYC ubiquitin ligase (FBW7) may prevent the MYC’s degradation, and the ubiquitin-specific protease USP28 has been shown to contribute to the MYC stabilization in neoplastic cells. RalA is a Ras effector, and its phosphorylation is also an important element of PP2A-mediated tumor suppression [30]. Altogether, MYC’s activity is regulated not only by CIP2A, but also by a number of other factors. The complexity of these regulatory systems would justify the intermediate, positive correlation we observed between the expression of the *KIAA1524* gene and the *MYC* gene (rho = 0.515; *p* = 0.0345) (Figure 2B).

Nevertheless, CIP2A is not the only factor capable of changing the PP2A’s activity. GFI-1 has been shown (growth factor independence-1) to inhibit the SGPP1 (sphingosine-1-phosphate phosphatase 1) gene transcription, leading to increased S1P (sphingosine-1-phosphate) synthesis. This resulted in a S1P/Ceramides ratio imbalance, triggering the PP2A inactivation and, as a consequence, c-Myc stabilization in the MM cells [33]. Such interactions must be considered when judging the prognostic roles of *KIAA1524* and *CIP2A*.

In the case of the 686 SNP of the *KIAA1524 (rs2278911)* gene, when cystein becomes replaced by tymin, the 229th amino acid coded by the gene changes from arginine (Arg) to glutamine (Gln) [37]. Such an alteration has been shown to lower the charge and change the surface properties of the CIP2A’s N-terminal domain, affecting the protein-to-protein interactions [34]. In addition, Preuss et al. found that certain modifications within the B subunit affected PP2A’s activity towards its targets [38]. A research paper by Wang et al. [35] revealed that, with its N-terminal (amino acids 1–560), CIP2A could bind directly to the B56α and B56γ subunits of the PP2A protein—both of which are tumor suppressor units—and that the region necessary for this was located between the amino acids 159 and 245 and positively charged. Moreover, the researchers found that changing the 230th (asparagine—neutrally charged) nucleotide to a negatively charged glutamic acid led to decreased binding of CIP2A to the B56α, and concluded that the B56 subunit was most likely to bind to a positively charged surface of the CIP2A [35].

In our study, the presence of the TT genotype of the KIAA1524 gene (rs2278911) had an independent prognostic value, with the afflicted patients being over seven times more likely to experience PFS and OS reduction compared to the carriers of other variants. Since the 686C > T alteration resulted in an exchange of a positively charged amino acid for a neutral one, it seems that in order to explain the TT genotype’s correlation with worse prognosis, we must search beyond the currently known direct CIP2A-PP2A interactions. In a paper published in 2014, three years before Wang’s publication, Puustinen et al. wrote that the mechanism in which CIP2A modulated the mTORC1-related PP2A activity was most likely that of an allosteric inhibitor, and stipulated that it was more likely to affect the substrate availability rather than the enzyme’s activity [39]. In 2021, Laine et al. discovered that poor clinical outcomes in basal-like breast cancer (BLBC) patients was related to high CIP2A expression and not to the alterations in its gene’s sequence. They also established that CIP2A expression was upregulated by the DNA-PF/CHK1 activity, TP53 inactivation and EGFR pathway activation [40]. As we have also observed a significantly lower expression of CIP2A among patients with the CC genotype, we would stipulate that that the consequences of indirect CIP2A-mediated PP2A modulation may outweigh the effects of the direct PP2A-binding, and that the most likely cause for the reported outcome–SNP correlation is, in fact, altered CIP2A expression. Our results would also align with a finding by Li et al., who speculated that Arg229Gln altered the CIP2A expression [37].

A number of drugs [41,42,43,44,45] have shown the ability to influence *KIAA1524*, CIP2A or their affected pathways. Bedewy and Elmaghraby observed that MM patients had higher CIP2A expression, which decreased during bortezomib treatment. Furthermore, higher CIP2A levels were correlated with a worse response to bortezomib, as well as with shorter PFS [45]. The research on *KIAA1524* and CIP2A as potential treatment targets is ongoing, but in order for it to progress, many obstacles must be overcome.

One of the biggest issues we are currently facing is a very convoluted net of CIP2A–PP2A interactions, which may be affected by a number of factors and has yet to be fully explored [34,46,47,48], as well as the partially contradicting data on the matter. A high CIP2A concentration has been found to serve as a negative prognostic factor in a number of human cancers [35], and CIP2A knockdown has been shown to inhibit the activity of MYC, E2F1 and Akt [30]; restrict tumor growth in oral cancer mice models [47] and result in decreased proliferation and increased apoptosis of human MM cells [48]. However, such results could not be achieved in all tumor types [46]. Additionally, although there seems to be a general consensus that CIP2A-mediated PP2A inhibition supports MM development, we did come across some research which pointed towards an oncogenic role of PP2A. In 2001 Kang and Chou found that PP2A regulated the ROI (reactive oxygen intermediates) levels within MM cells, which promoted the expression of IL-6 and BCL-2, thus supporting the MM’s development [49]. Three years later, Mitsuthashi et al. demonstrated that PP2A prevented the degradation of the glycoprotein 130 (also known as IL-6 signal transducer). The researchers observed that bortezomib-mediated gp130 degradation provoked the MM cells’ apoptosis by decreasing their sensitivity to the IL-6 [50]. In 2021, Slomp et al. found that PP2A reversed the dephosphorylated and stabilized MCL1 (induced myeloid leukemia cell differentiation protein) in MM [45].

Based on data from the CoMMpass, we identified 30 candidate genes, the regulation of expression of which may be related to *KIAA*: *AC069499.1*, *MYH15*, *HNRNPR*, *KIF11*, *CENPI, USP1, CEP78, AMMECR1, RRM1, PLK4, TMPO, SMC4, SGO1, CEP55*, *BUB1*, *OSBPL11*, *KIF4A*, *CAPG*, *SPAG5*, *INCENP*, *POLA1*, *CDK1*, *ZWINT*, *SMC2*, *PCNA*, *SPATA5, NCAPG2*, *GPSM2*, *NCAPH* and *TOP2A.* Disturbances in their expression have been the subject of many studies on the pathogenesis of solid tumors, e.g., lung, breast, endometrial, stomach and colon cancer [51,52,53,54,55,56,57,58,59,60,61,62,63,64,65,66,67,68].

Interestingly, these include genes encoding centromeric proteins (*CENPI*) [51]; centrosomal proteins (*CEP55*, *CEP78*) [52,62]; motor proteins (*MYH15*, *KIF11*) [53,54] involved in the construction of the karyokinetic spindle or proteins regulating cell division processes (*PLK4, SGO1, BUB1, KIF4A, NCAPG, SPAG5, INCENP, CDK1, ZWINT, NCAPG2, GPSM2, NCAPH* and *TOP2A*) [55,60,63,66,67,68,69,70,71,72,73]; and genes encoding proteins involved in DNA repair processes (*USP1, RRM1, KIF4A, POLA1* and *PCNA*) [58,59,65,74,75], which are defined as oncogenes (*SPAG5, SMC2*) [67,76].

In the context of multiple myeloma, the expression of the *HNRNPA2B1*, *USP1*, *RRM1*, *SPAG5*, *PCNA* and *TOP2A* genes has been studied. It has been proven that *HNRNPA2B1* increases the stability of mRNA transcripts for interleukin enhancer-binding factor 3 (ILF3), and its overexpression induces cell proliferation through the AKT3 kinase. Increased *HNRNPA2B1* gene expression is associated with an unfavorable prognosis [57]. The USP1 protein leads to regulated DNA repair through deubiquitination and homologous recombination pathways, and inhibits cell differentiation by stabilizing tumor-promoting inhibitors of DNA-binding proteins. Inhibition of *USP1* has been shown to reduce myeloma cell viability, inhibit the growth of multiple myeloma cells and overcome bortezomib resistance [58]. Higher expression of the *RRM1* gene is associated with shorter overall survival. A blockade of the *RRM1* gene results in the upregulation of DNA damage response genes and p53-regulated genes, thereby inhibiting the growth and promoting apoptosis of myeloma cells and cells of the bone marrow microenvironment [59]. *SPAG5* gene expression correlates with myeloma cell malignancy, and *SPAG5* overexpression is associated with poorer clinical outcomes. In vitro, knockdown of the *SPAG5* gene leads to anti-MM effects, cell growth arrest and apoptosis via the PI3K/AKT/mTOR pathway [67]. The *PCNA* gene encodes a multifunctional protein which is essential for DNA replication and repair and is overexpressed in myeloma cells. *PCNA* inhibition induces apoptosis and sensitizes cells to melphalan, a common anti-myeloma drug [75]. *TOP2A* has been demonstrated to be another potential target for anti-myeloma therapy. It has been observed that overexpression of TOP2A topoisomerase in myeloma cells is associated with resistance to proteasome inhibitors [77].

## 5. Conclusions

The varied clinical course of MM, differences in the efficacy of specific therapeutic strategies, and different lengths of time needed for chemoresistance development imply a need to identify risk stratification factors that would enable the personalization of therapy and the improvement of treatment outcomes. For this purpose, we can use prognostic markers, which assess the risk of an unfavorable course of a disease and its recurrence, as well as predictive factors, which determine the probability of obtaining remission with a specific treatment method. Although our results are preliminary, they indicate that the assessment of the SNP 686C > T of the *KIAA1524* gene may be a useful biomarker for determining the prognosis of MM patients.

## Figures and Tables

**Table 1:** Baseline characteristics of the study group.

Variable	*n* = 128 (100%)
Sex	
Men	64 (50%)
Women	64 (50%)
Age	
<65	58 (45.3%)
≥65	70 (54.7%)
Myeloma type	
IgG	72 (56.2.%)
IgA	29 (22.7%)
Light chains	25 (19.5%)
Non-secretory	1 (0.8%)
Plasmocytoma	1 (0.8%)
Light chain type	
Kappa	80 (63%)
Lambda	47 (37%)
No data: *n* = 1	
Durie–Salmon stage	
I	12 (9.4%)
II	16 (12.5%)
III	100 (78.1%)
ISS stage	
1	33 (25.8%)
2	36 (28.1%)
3	59 (46.1%)
Renal function	
A—creatinine < 2 mg/dL	102 (79.7%)
B—creatinine ≥ 2 mg/dL	26 (20.3%)
Performance status	
0	11 (8.6%)
1	49 (38.3%)
2	51 (39.8%)
3	15 (11.7%)
4	2 (1.6%)
Body weight loss	
No	47 (50.5%)
Yes	46 (49.5%)
No data: *n =* 35	
5%	14 (30.4%)
10%	32 (69.6%)
Anemia grade before treatment (WHO)	
Absent or I°	65 (50.8%)
II°, III° or IV°	63 (49.2%)
Treatment protocol	
CTD	27 (21.1%)
V(C)D	58 (45.3%)
VTD	43 (33.6%)
AHSCT	82 (64.1%)
No	46 (35.9%)
Yes	
*del 17p/TP53*	
Absent	65 (74.7%)
Present	22 (25.3%)
No data: *n* = 41	
*t(4;14) IGH/FGFR3*	
Absent	75 (85.2%)
Present	13 (14.8%)
No data: *n* = 40	
*t(11;14) IGH/CCND1*	
Absent	87 (88.8%)
Present	11 (11.2%)
No data: *n* = 30	
*t(14;16) IGH/MAF*	
Absent	82 (83.2%)
Present	6 (6.8%)
No data: *n* = 40	
Other *IGH* rearrangement	
Absent	84 (85.7%)
Present	14 (14.3%)
No data: *n* = 30	
amp *1q21/CKS1B*	
Absent	11 (47.8%)
Present	12 (52.2%)
No data: *n* = 105	

CTD—cyclophosphamide, thalidomide, dexamethsone; ISS—Multiple Myeloma International Staging System; WHO—World Health Organization; V(C)D—bortezomib, (cyclophosphamide), dexamethasone; VTD—bortezomib, thalidomide, dexamethasone.

## Data Availability

The research data are available from the corresponding author upon reasonable request. The data are not publicly available due to the fact that the data sheet contains information that exceeds the scope of this study, and which may be used for other research papers in the future.

## Supplementary Materials

The following supporting information can be downloaded at: https://www.mdpi.com/article/10.3390/cells12071029/s1, Table S1: Distribution of KIAA1524 genotypes depending on selected demographic, clinical and molecular variables. Table S2: Chemotherapy response depending on selected demographic, clinical and molecular variables. Table S3: Survival of MM patients depending on selected demographic, clinical and molecular variables.
