# Supplementary material for: The TT Genotype of the KIAA1524 rs2278911 Polymorphism Is Associated with Poor Prognosis in Multiple Myeloma
Source: Cells. 2023 Mar 28;12(7):1029. doi: 10.3390/cells12071029 (PMC10093279; doi:10.3390/cells12071029)
Supplement: Supplementary file 1 [file cells-12-01029-s001.zip › cells-2186509-supplementary.pdf]

Supplementary Materials

Supplementary Table S1. Distribution of *KIAA1524* genotypes depending on selected demographic, clinical and molecular varables.

Variable	*KIAA1524* genotypes			*p*
	CC	CT	TT	
Sex				
Men	47 (73.4%)	14 (21.9%)	3 (4.7%)	0.6965
Women	51 (79.7%)	11 (17.2%)	2 (3.1%)	
Diagnosis				
Light chain disease	18 (72%)	6 (24%)	1 (4%)	0.8176
MM with a monoclonal component, Non-secretory MM, plasmacytoma	80 (77.7%)	19 (18.4%)	4 (3.9%)	
Monoclonal protein class				
IgA	25 (73.5%)	8 (23.5%)	1 (2.9%)	0.5337
IgG	56 (78.9%)	11 (15.5%)	4 (5.6%)	
No data: n=23				
Light chain type				
Lambda	36 (76.6%)	8 (17%)	3 (6.4%)	0.5284
Kappa	62 (77.5%)	16 (20%)	2 (2.5%)	
No data: n=1				
Durie Salmon stage				
III	89 (76.7%)	23 (19.8%)	4 (3.4%)	0.6962
I, II	9 (75%)	2 (16.7%)	1 (8.3%)	
ISS stage				
3	41 (70.7%)	14 (24.1%)	3 (5.2%)	0.3560
1, 2	57 (81.4%)	11 (15.7%)	2 (2.9%)	
Renal function				
B	19 (73.1%)	5 (19.2%)	2 (7.7%)	0.5351
A	79 (77.5%)	20 (19.6%)	3 (2.9%)	
Stage of chronic kidney disease				
G3a/G3b/G4/G5D	34 (66.7%)	14 (27.5%)	3 (5.9%)	0.1113
G1/G2	55 (83.3%)	9 (13.6%)	2 (3.0%)	
Body weight loss before treatment				
Yes	24 (75%)	6 (18.8%)	2 (6.2%)	0.6291
No	11 (78.6%)	3 (21.4%)	0 (0%)	
No data: n=35				

Anaemia grade before treatment (WHO)<br>Absent or I°<br>II°, III° or IV°	52 (80%)<br>46 (73%)	12 (18.5%)<br>13 (20.6%)	1 (1.5%)<br>4 (6.3%)	0.3368
Haemoglobin<br>Low<br>Normal	83 (75.5%)<br>15 (83.3%)	22 (20%)<br>3 (16.7%)	5 (4.5%)<br>0 (0%)	0.5970
Platelets<br>Low<br>Normal	47 (73.4%)<br>51 (79.7%)	14 (21.9%)<br>11 (17.2%)	3 (4.7%)<br>2 (3.1%)	0.6965
Albumins<br>Low<br>Normal	36 (75%)<br>62 (77.5%)	10 (20.8%)<br>15 (18.8%)	2 (4.2%)<br>3 (3.7%)	0.9491
CRP<br>High<br>Normal	33 (75%)<br>65 (77.4%)	8 (18.2%)<br>17 (20.2%)	3 (6.8%)<br>2 (2.4%)	0.4632
LDH<br>High<br>Normal	18 (78.3%)<br>80 (76.2%)	5 (21.7%)<br>20 (19%)	0 (0%)<br>5 (4.8%)	0.5555
Calcium<br>High<br>Normal	20 (64.5%)<br>78 (80.4%)	10 (32.3%)<br>15 (15.5%)	1 (3.2%)<br>4 (4.1%)	0.1215
B2M<br>High<br>Normal	87 (76.3%)<br>11 (78.6%)	23 (20.2%)<br>2 (14.3%)	4 (3.5%)<br>1 (7.1%)	0.7221
Creatinine<br>High<br>Normal	36 (70.6%)<br>62 (80.5%)	13 (25.5%)<br>12 (15.6%)	2 (3.9%)<br>3 (3.9%)	0.3797
eGFR<br>Low<br>Normal	64 (74.4%)<br>34 (81%)	17 (19.8%)<br>8 (19%)	5 (5.8%)<br>0 (0%)	0.2716
del 17p/TP53<br>Absent<br>Present<br>*No data: n=41*	50 (76.9%)<br>16 (72.7%)	11 (16.9%)<br>5 (22.7%)	4 (6.2%)<br>1 (4.5%)	0.8133
t(4;14) IGH/FGFR3<br>Absent<br>Present	60 (80%)<br>7 (53.8%)	13 (17.3%)<br>3 (23.1%)	2 (2.7%)<br>3 (23.1%)	0.0095*

No data: n=40				
t(14;16) IGH/MAF				
Absent	63 (76.8%)	14 (17.1%)	5 (6.1%)	0.5340
Present	4 (66.7%)	2 (33.3%)	0 (0%)	
No data: n=40				
t(11;14) IGH/CCND1				
Absent	67 (77%)	16 (18.4%)	4 (4.6%)	0.8146
Present	8 (72.7%)	2 (18.2%)	1 (9.1%)	
No data: n=30				
Other IGH rearrangement				
Absent	64 (76.2%)	16 (19%)	4 (4.8%)	0.8649
Present	11 (78.6%)	2 (14.3%)	1 (7.1%)	
No data: n=30				
amp 1q21/ CKS1B				
Absent	8 (72.7%)	2 (18.2%)	1 (9.1%)	0.4134
Present	11 (91.7%)	1 (8.3%)	0 (0%)	
No data: n=105				

Supplementary Table S2. Chemotherapy response depending on selected demographic, clinical and molecular variables.

**Variable**	**2nd cycle [n=122]**		**OR [95%CI] *p***	**4th cycle [n=110]**		**OR [95%CI] *p***	**6th cycle [n=98]**		**OR [95%CI] *p***	**8th cycle [n=38]**		**OR [95%CI] *p***
	**No response**	**Response**		**No response**	**Response**		**No response**	**Response**		**No response**	**Response**	
**Sex**			1.39			6.36			2.38			1.46
Men	8 (9.8%)	55 (90.2%)	[0.44-4.25]	6 (10.7%)	50 (89.3%)	[0.74-54.71]	4 (8.7%)	42 (9.13%)	[0.41-13.65]	3 (18.8%)	13 (81.2%)	[0.25-8.40]
Women	6 (13.1%)	53 (86.9%)	0.5711	1 (1.9%)	53 (98.1%)	0.0920	2 (3.8%)	50 (96.2%)	0.3302	3 (13.6%)	19 (86.4%)	0.6706
**Age**			0.86			0.62			0.81			2.27
≥65	7 (10.8%)	58 (89.2%)	[0.28-2.62]	3 (5.1%)	56 (94.9%)	[0.13-2.95]	3 (5.6%)	51 (94.4%)	[0.15-4.19]	5 (18.5%)	22 (81.5%)	[0.23-22.07]
<65	7 (12.3%)	50 (87.7%)	0.7940	4 (7.8%)	47 (92.2%)	0.5574	3 (6.8%)	41 (93.2%)	0.7957	1 (9.1%)	10 (90.9%)	0.4791
**Diagnosis**	12 (12%)	88 (88%)	1.23	7 (7.5%)	86 (92.5%)	2.86		78 (94%)	2.03	5 (16.1%)	26 (83.9%)	1.15

IgG or IgA	2 (10%)	18 (90%)	[0.25-5.96]	0 (0%)	16 (100%)	[0.15-52.56]	5 (6%)	14 (100%)	[0.11-38.77]	1 (14.3%)	6 (85.7%)	[0.11-11.78]
Light chain disease			0.7995			0.4790	0 (0%)		0.6375			0.9039
N/A: n=2												
**Monoclonal protein class**			2.18			2.74			0.59			0.20
IgG	10 (13.9%)	62 (86.1%)	[0.45-10.61]	6 (9.2%)	59 (90.8%)	[0.31-23.94]	3 (5.1%)	56 (94.9%)	[0.09-3.77]	2 (9.1%)	20 (90.9%)	[0.03-1.49]
IgA	2 (6.9%)	27 (93.1%)	0.3356	1 (3.6%)	27 (96.4%)	0.3606	2 (8.3%)	22 (91.7%)	0.5765	3 (33.3%)	6 (66.7%)	0.1163
N/A: n=27												
**Light chain type**			1.00			2.67			3.10			0.83
Lambda	5 (11.6%)	38 (88.4%)	[0.31-3.23]	4 (10.5%)	34 (89.5%)	[0.56-12.60]	3 (9.1%)	30 (90.9%)	[0.49-19.55]	2 (14.3%)	12 (85.7%)	[0.13-5.26]
Kappa	9 (11.5%)	69 (88.5%)	0.9883	3 (4.2%)	68 (95.8%)	0.2156	2 (3.1%)	62 (96.9%)	0.2285	4 (16.7%)	20 (83.3%)	0.8462
N/A: n=1												
**Durie Salmon stage**			1.05<br>[0.27-4.06]			0.72<br>[0.13-3.95]			0.67<br>[0.11-3.88]			1.05<br>[0.16-6.64]
III	11 (11.6%)	84 (88.4%)	0.9463	5 (5.9%)	80 (94.1%)	0.7041	4 (5.5%)	69 (94.5%)	0.6519	4 (16%)	21 (84%)	0.9606
I, II	3 (11.1%)	24 (88.9%)		2 (8%)	23 (92%)		2 (8%)	23 (92%)		2 (15.4%)	11 (84.6%)	
**ISS stage**			1.87			1.01			1.36			1.28
3	8 (15.1%)	45 (84.9%)	[0.61-5.75]	0 (0%)	4 (100%)	[0.21-4.72]	0 (0%)	2 (100%)	[0.26-7.10]	0 (0%)	1 (100%)	[0.22-7.37]
1, 2	6 (8.7%)	63 (91.3%)	0.2771	1 (5.6%)	17 (94.4%)	0.9943	3 (20%)	12 (80%)	0.7161	1 (25%)	3 (75%)	0.7779
**Renal function**	4 (17.4%)	19 (82.6%)	1.87	2 (9.5%)	19 (90.5%)	1.77	1 (5.6%)	17 (94.4%)	0.88	3 (33.3%)	6 (66.7%)	4.33

B	10 (10.1%)	89 (89.9%)	[0.53-6.61]	5 (5.6%)	84 (94.4%)	[0.32-9.81]	5 (6.2%)	75 (93.7%)	[0.09-8.05]	3 (10.3%)	26 (89.7%)	[0.69-27.01]
A			0.3290			0.5144			0.9116			0.1163
**Stage of chronic kidney disease**			1.08			0.55			1.45			5.71
G3a/G3b/G4/G5D	6 (13%)	40 (87%)	[0.35-3.37]	2 (4.9%)	39 (95.1%)	[0.10-3.00]	2 (5.4%)	35 (94.6%)	[0.19-10.84]	5 (26.3%)	14 (73.7%)	[0.59-54.96]
G1/G2	8 (12.1%)	58 (87.9%)	0.8846	5 (8.5%)	54 (91.5%)	0.4934	2 (3.8%)	51 (96.2%)	0.7131	1 (5.9%)	16 (94.1%)	0.1313
N/A: n=11												
**Performance status**			3.95			1.41			0.52			0.78
			[1.04-14.95]			[0.30-6.63]			[0.09-2.99]			[0.13-4.59]
2-4	11 (17.5%)	52 (82.5%)		4 (7.4%)	50 (92.6%)		2 (4.3%)	45 (95.7%)		3 (14.3%)	18 (85.7%)	
			0.0432*			0.6610			0.4658			0.7779
0, 1	3 (5.1%)	56 (94.9%)		3 (5.4%)	53(94.6%)		4 (7.8%)	47 (92.2%)		3 (17.6%)	14 (82.4%)	
**Treatment protocol (1)**			5.35			3.31			0.31			0.60
			[1.66-17.22]			[0.68-16.05]			[0.02-5.75]			[0.06-5.93]
CTD	7 (29.2%)	17 (70.8%)		3 (13.6%)	19 (86.4%)		0 (0%)	18 (100%)		1 (11.1%)	8 (88.9%)	
			0.0049*			0.1364			0.4317			0.6621
V(C)D, VTD	7 (7.1%)	91 (92.9%)		4 (4.5%)	84 (95.5%)		6 (7.5%)	74 (92.5%)		5 (17.2%)	24 (82.8%)	
**Treatment protocol (2)**			0.05			0.26			1.79			1.08
			[0.003-0.93]			[0.03-2.26]			[0.34-9.36]			[0.10-11.32]
VTD	0 (0%)	42 (100%)		1 (2.4%)	40 (97.6%)		3 (8.3%)	33 (91.7%)		1 (16.7%)	5 (83.3%)	
			0.0443*			0.2235			0.4917			0.9488
CTD, V(C)D	14 (17.5%)	66 (82.5%)		6 (8.7%)	63 (91.3%)		3 (4.8%)	59 (95.2%)		5 (15.6%)	27 (84.4%)	
**Body weight loss**	6 (14%)	37 (86%)	0.77	4 (10.5%)	37 (94.9%)	2.18	1 (3%)	32 (97%)	0.56	2 (15.4%)	11 (84.6%)	0.79
**before treatment**	8 (17.4%)	38 (82.6%)	[0.24-2.43]	2 (5.1%)	32 (89.5%)	[0.37-12.65]	2 (5.3%)	36 (94.7%)	[0.05-6.50]	3 (18.8%)	13 (81.2%)	[0.11-5.60]

Yes			0.6568			0.3865			0.6449			0.8117
No												
N/A: n=35												
**Anaemia before treatment (WHO)**			7.13			3.24			1.05			0.33
Yes	14 (13.9%)	87 (86.1%)	[0.41-124.23]	7 (7.6%)	85 (92.5%)	[0.18-59.37]	5 (6.2%)	76 (93.8%)	[0.11-9.63]	5 (14.3%)	30 (85.7%)	[0.02-4.40]
No	0 (0%)	21 (100%)	0.1781	0 (0%)	18 (100%)	0.4273	1 (5.9%)	16 (94.1%)	0.9638	1 (33.3%)	2 (66.7%)	0.4040
**Platelets**			1.33			1.15			9.22			7.50
Low	2 (14.3%)	12 (85.7%)	[0.26-6.69]	1 (7.1%)	13 (92.9%)	[0.13-10.37]	3 (25%)	9 (75%)	[1.61-52.64]	2 (50%)	2 (50%)	[0.81-69.08]
Normal	12 (11.1%)	96 (88.9%)	0.7266	6 (6.2%)	90 (93.7%)	0.8983	3 (3.5%)	83 (96.5%)	0.0124*	4 (11.8%)	30 (88.2%)	0.0753
**Albumins**			2.56			1.52			0.39			2.20
Low	8 (17.8%)	37 (82.2%)	[0.83-7.92]	3 (8.1%)	34 (91.9%)	[0.32-7.19]	1 (3.1%)	61 (96.9%)	[0.04-3.52]	3 (23.1%)	10 (76.9%)	[0.38-12.87]
Normal	6 (7.8%)	71 (92.2%)	0.1034	4 (5.5%)	69 (94.5%)	0.5958	5 (7.6%)	31 (92.4%)	0.4040	3 (12%)	22 (88%)	0.3816
**CRP**			3.32			6.70			0.57			0.44
High	8 (20.5%)	31 (79.5%)	[1.06-10.33]	5 (15.2%)	28 (84.8%)	[1.23-36.52]	1 (4%)	24 (96%)	[0.06-5.09]	1 (9.1%)	10 (90.9%)	[0.04-4.27]
Normal	6 (7.2%)	77 (92.8%)	0.0391*	2 (2.6%)	75 (97.4%)	0.0280*	5 (6.8%)	68 (93.2%)	0.6123	5 (18.5%)	22 (81.5%)	0.4791
**LDH**			1.47			0.66			2.87			0.94
High	2 (15.4%)	11 (84.6%)	[0.29-7.43]	0 (0%)	9 (100%)	[0.03-12.54]	1 (14.3%)	6 (85.7%)	[0.29-28.62]	0 (0%)	2 (100%)	[0.04-21.95]
Normal	12 (11%)	97 (89%)	0.6416	7 (6.9%)	94 (93.1%)	0.7842	5 (5.5%)	86 (94.5%)	0.3697	6 (16.7%)	30 (83.3%)	0.9685
**Calcium**	5 (16.7%)	25 (83.3%)	1.84	1 (3.7%)	26 (96.3%)	0.49	3 (13.6%)	19 (86.4%)	3.84	1 (10%)	9 (90%)	0.51
High	9 (9.8%)	83 (90.2%)	[0.57-6.01]	6 (7.2%)	77 (92.8%)	[0.06-4.29]	3 (3.9%)	73 (96.1%)	[0.72-20.57]	5 (17.9%)	23 (82.1%)	[0.05-5.00]

Normal			0.3097			0.5224			0.1159			0.5642
**B2M**			0.75			0.33			0.75			0.33
High	12 (14.3%)	96 (85.7%)	[0.15-3.76]	5 (14.3%)	91 (94.8%)	[0.06-1.89]	5 (5.9%)	80 (94.1%)	[0.08-6.98]	5 (14.3%)	30 (85.7%)	[0.02-4.40]
Normal	2 (11.1%)	12 (88.9%)	0.7266	2 (5.2%)	12 (85.7%)	0.2131	1 (7.7%)	12 (92.3%)	0.8005	1 (33.3%)	2 (66.7%)	0.4040
**Creatinine**			1.33			0.71		32 (97%)	0.37			3.33
High	6 (13.3%)	39 (86.7%)	[0.43-4.10]	2 (5.1%)	37 (94.9%)	[0.13-3.86]	1 (3%)	60 (92.3%)	[0.04-3.35]	4 (25%)	12 (75%)	[0.53-21.03]
Normal	8 (10.4%)	69 (89.6%)	0.6234	5 (7%)	66 (93%)	0.6952	5 (7.7%)		0.3799	2 (9.1%)	20 (90.9%)	0.2002
**eGFR**			0.94			1.29			0.97			3.82
Low	9 (11.3%)	71 (88.7%)	[0.29-3.00]	5 (6.8%)	68 (93.2%)	[0.24-6.97]	4 (6.1%)	62 (93.9%)	[0.17-5.58]	6 (19.4%)	25 (80.6%)	[0.19-75.99]
Normal	5 (11.9%)	37 (88.1%)	0.9142	2 (5.4%)	35 (94.6%)	0.7699	2 (6.3%)	30 (93.7%)	0.9707	0 (0%)	7 (100%)	0.3792
**del 17p/TP53**			0.21			2.03			12.46			0.62
Present	0 (0%)	21 (100%)	[0.01-3.87]	2 (9.5%)	19 (90.5%)	[0.32-13.11]	3 (18.8%)	13 (81.2%)	[1.20-129.73]	1 (14.3%)	6 (85.7%)	[0.06-6.80]
Absent	6 (9.4%)	58 (90.6%)	0.2936	3 (4.9%)	58 (95.1%)	0.4547	1 (1.8%)	54 (98.2%)	0.0348*	4 (21.1%)	15 (78.9%)	0.6996
No data: n=41												
**t(4;14)**												
**IGH/FGFR3**			1.25			5.11			2.18			2.37
Present	1 (8.3%)	11 (91.7%)	[0.13-11.77]	2 (18.2%)	9 (81.8%)	[0.75-34.84]	1 (10%)	9 (90.%)	[0.20-23.36]	1 (33.3%)	2 (66.7%)	[0.17-33.00]
Absent	5 (6.8%)	69 (93.2%)	0.8427	3 (4.2%)	69 (95.8%)	0.0957	3 (4.8%)	59 (95.2%)	0.5179	4 (17.4%)	19 (82.6%)	0.5194
No data: n=40												
**t(11;14)**	2 (18.2%)	9 (81.8%)	2.92	2 (20%)	8 (80%)	6.42	4 (44.4%)	5 (55.6%)	55.20	2 (66.7%)	1 (33.3%)	16.00
**IGH/CCND1**	6 (7.1%)	79 (92.9%)	[0.51-16.71]	3 (3.7%)	77 (96.2%)	[0.93-44.28]	1 (1.4%)	69 (98.6%)	[5.15-591.63]	3 (11.1%)	24 (88.9%)	[1.09-234.26]

Present<br>Absent<br>*No data: n=30*			0.2272			0.0593#			0.0009*			0.0429*
**t(14;16) IGH/MAF**<br>Present<br>Absent<br>*No data: n=30*	0 (0%)<br>8 (8.5%)	2 (100%)<br>86 (91.5%)	2.03<br>[0.09-45.96]<br>0.6550	0 (0%)<br>5 (5.7%)	2 (100%)<br>83 (94.3%)	3.04<br>[0.13-71.33]<br>0.4904	0 (0%)<br>5 (6.5%)	2 (100%)<br>72 (93.5%)	2.64<br>[0.11-62.00]<br>0.5474	0 (0%)<br>5 (17.9%)	2 (100%)<br>23 (82.1%)	0.85<br>[0.03-20.45]<br>0.9227
**Other IGH rearrangement**<br>Present<br>Absent<br>*No data: n=30*	1 (7.1%)<br>7 (8.5%)	13 (92.9%)<br>75 (91.5%)	0.82<br>[0.09-7.26]<br>0.8618	0 (0%)<br>5 (6.6%)	14 (100%)<br>71 (93.4%)	0.45<br>[0.02-8.56]<br>0.5939	0 (0%)<br>5 (7.6%)	13 (100%)<br>61 (92.4%)	0.41<br>[0.02-7.94]<br>0.5587	2 (33.3%)<br>3 (12.5%)	4 (66.7%)<br>21 (87.5%)	3.50<br>[0.43-28.14]<br>0.2388
**Amp 1q21/CKS1B**<br>Present<br>Absent<br>*No data: n=105*	0 (0%)<br>0 (0%)	13 (100%)<br>9 (100%)	N/d	1 (7.7%)<br>0 (0%)	12 (92.3%)<br>9 (100%)	2.28<br>[0.08-62.44]<br>0.6255	2 (18.2%)<br>1 (16.7%)	9 (81.8%)<br>5 (83.3%)	1.11<br>[0.08-15.53]<br>0.9376	0 (0%)<br>1 (100%)	4 (100%)<br>0 (0%)	0.04<br>[0.0004-2.83]<br>0.1361
***KIAA1524* genotype (686G>A, rs2278911)**	7 (7.4%)<br>7 (25.9%)	88 (92.6%)<br>20 (74.1%)	0.23<br>[0.07-0.72]<br>0.0119*	4 (4.7%)<br>3 (12.5%)	82 (95.3%)<br>21 (87.5%)	0.34<br>[0.07-1.64]<br>0.1803	3 (3.9%)<br>3 (14.3%)	74 (96.1%)<br>18 (85.7%)	0.24<br>[0.04-1.31]<br>0.0993	4 (13.8%)<br>2 (22.2%)	25 (86.2%)<br>7 (77.8%)	0.56<br>[0.08-3.72]<br>0.5483

CC<br>CT r CT												
***KIAA1524* genotype (686G>A, rs2278911)**<br>TT<br>CC r CT	2 (50%)<br>12 (10.2%)	2 (50%)<br>106 (89.8%)	8.33<br>[1.14-68.54]<br>0.0372*	2 (50%)<br>5 (4.7%)	2 (50%)<br>101 (95.3%)	20.20<br>[2.34-174.44]<br>0.0063*	1 (33.3%)<br>5 (5.3%)	2 (66.7%)<br>90 (94.7%)	9.00<br>[0.69-116.87]<br>0.0930	1 (50%)<br>5 (13.9%)	1 (50%)<br>31 (86.1%)	6.20<br>[0.33-115.92]<br>0.2220

Abbreviations: B2M – beta-2-microglobulin, CI – confidence interval, CRP – C-reactive protein, eGFR – estimated glomerular filtration rate, ISS - Multiple Myeloma International Staging System, LDH - lactate dehydrogenase, *Me* – median, N/d – not determined, OR – odds ratio, *p* - statistical significance, WHO -World Health Organization. * - statistically significant result, # - a trend toward significance.

Supplementary Table S3. Survival of MM patients depending on selected demographic, clinical and molecular variables.

Variable	Progression free survival			Overall survival		
	Univariable		Multivariable	Univariable		Multivariable
	mPFS (months)	HR (95% CI) *p*	HR (95% CI) *p*	mOS (months)	HR (95% CI) *p*	HR (95% CI) *p*
	25			43		
Sex						
Men	25	1.65 (1.03-2.63)	1.73 (0.91-3.27)	34	1.35 (0.77-2.36)	1.46 (0.65-3.27)
Women	27	0.0325*	0.0956	46	0.2889	0.3613
Age						
≥65	17	1.49 (0.34-2.38)	0.72 (0.33-1.54)	38	1.35 (0.77-2.36)	0.89 (0.40-2.02)
<65	30	0.0930	0.3949	45	0.3056	0,7908
Diagnosis						
Light chain disease	24	1.03 (0.55-1.95)	0.92 (0.40-2.13)	28	2.37 (1.04-5.40)	3.52 (1.45-8.55)
IgA, IgG	25	0.9168	0.8554	47	0.0057*	0.0056*
N/A: n=2						
Monoclonal protein class						
IgA	24	1.02 (0.58-1.79)	1.38 (0.62-3.09)	45	1.44 (0.70-2.98)	0.55 (0.20-1.46)
IgG	25	0.9432	0.4294	47	0.2841	0.2313
N/A: n=27						
Light chain type						
Lambda	17	1.27 (0.77-2.09)	1,41 (0.76-2.62)	33	1.63 (0.89-3.00)	1.09 (0.47-2.48)
Kappa	27	0.3169	0.2821	43	0.0825	0.8450
No data: n=1						
Durie-Salmon stage						
III	25	0.84 (0.47-1.49)	0.50 (0,20-1.25)	41	1.07 (0.54-2.11)	0.79 (0.24-2.60)
I, II	17	0.5241	0,1423	45	0.8492	0.6963
ISS stage						
3	17	1.74 (1.08-2.82)	1.16 (0.58-2.31)	34	2.03 (1.13-3.65)	2.11 (1.20-3.73)
1, 2	34	0.0153*	0,6704	45	0.0099*	0.0101*
Renal function						
B	24	1.24 (0.68-2.24)	0.69 (0.31-1.57)	28	1.36 (0.65-2.85)	0.61 (0.22-1.71)
A	25	0.4431	0.3847	43	0.3572	0.3539
Stage of chronic kidney disease						
G3a/G3b/G4/G5D	17	1.85 (0.12-3.06)	1.21 (0.58-2.53)	43	1.78 (0.99-3.20)	0.85 (0.35-2.01)
G1/G2	34	0.0092*	0.6126	NR	0.0436*	0.7071
N/A: n=11						
Performance status						
2-4	17	1.60 (1.01-2.55)	0.85 (0.42-1.73)	33	2.01 (1.15-3.53)	2.19 (0.98-4.88
0, 1	34	0.0479*	0.6504	47	0.0144*	0.0566[#]
Treatment protocol (1)						
CTD	24	1.18 (0.69-2.03)	0.94 (0.45-1.95)	33	0.95 (0.52-1.73)	1.24 (0.48-3.22)
V(C)D, VTD	25	0.5006	0,8662	41	0.8488	0.6646
Treatment protocol (2)						
VTD	36	0.56 (0.34-0.92)	0.96 (0.44-2.10)	34	1.08 (0.58-2.01)	1.29 (0.50-3.35)
CTD, V(C)D	18	0.0352*	0.9143	47	0.7904	0.6043
AHSCT						
Yes	42	0.37 (0.23-0.59)	0.48 (0.25-0.93)	45	0.53 (0.30-0.93)	0.88 (0.36-2.22)
No	16	0.0002*	0.0308*	38	0.0386*	0.7937

Body weight loss before treatment<br>Yes<br>No<br>*No data: n=35*	18<br>34	1.46 (0.87-2.45)<br>0.1471	1,18 (0,55-2,52)<br>0,6672	33<br>46	1.36 (0.73-2.52)<br>0.3181	1.55 (0.63-3.83)<br>0.3412
Anaemia before treatment (WHO)<br>Yes<br>No	24<br>NR	2.40 (1.35-4.26)<br>0.0196*	1,46 (0,54-4,00)<br>0,4593	47<br>33	2.43 (1.28-4.63)<br>0.0280*	3.29 (0.94-11.94)<br>0.0722
Platelets<br>Low<br>Normal	10<br>26	2.08 (0.95-4.56)<br>0.0127*	3.07 (1.41-6.71)<br>0.0051*	24<br>43	1.54 (0.67-3.58)<br>0.2288	2.21 (0.81-6.06)<br>0.1249
Albumins<br>Low<br>Normal	14<br>34	2.29 (1.37-3.84)<br>0.0002*	1.50 (0.79-2.83)<br>0.2186	30<br>47	2.28 (1.22-4.26)<br>0.0023*	4.08 (1.71-9.77)<br>0.0016*
CRP<br>High<br>Normal	24<br>26	1.29 (0.78-2.13)<br>0.2929	0.88 (0.42-1.82)<br>0.7343	38<br>46	1.42 (0.77-2.64)<br>0.2265	2.14 (0.83-5.52)<br>0.1159
LDH<br>High<br>Normal	12<br>25	1.95 (0.85-4.48)<br>0.0344*	2.59 (1.01-6.62)<br>0.0487*	22<br>43	1.79 (0.66-4.88)<br>0.1442	2.11 (0.59-7.55)<br>0.2534
Calcium<br>High<br>Normal	24<br>25	1.36 (0.78-2.37)<br>0.2350	1.71 (0.83-3.54)<br>0.1464	28<br>46	1.63 (0.82-3.22)<br>0.1094	1.30 (0.54-3.17)<br>0.5589
B2M<br>High<br>Normal	25<br>NR	2.58 (1.37-4.84)<br>0.0278*	1.28 (0.43-3.81)<br>0.6562	34<br>52	2.73 (1.35-5.53)<br>0.0373*	1.47 (0.40-5.36)<br>0.5639
Creatinine<br>High<br>Normal	17<br>30	1.64 (1.01-2.68)<br>0.0315*	1.09 (0.54-2.18)<br>0.8135	34<br>47	1.74 (0.97-3.13)<br>0.0466*	1.38 (0.59-3.22)<br>0.4638
eGFR<br>Low<br>Normal	24<br>39	1.44 (0.88-2.36)<br>0.1735	0.59 (0.28-1.27)<br>0.1808	38<br>45	1.30 (0.72-2.37)<br>0.4037	1.59 (0.62-4.08)<br>0.3394
del 17p/TP53<br>Present<br>Absent<br>*No data: n=41*	15<br>25	1.84 (0.92-3.66)<br>0.0391*	2.25 (1.17-4.31)<br>0.0150*	30<br>46	1.68 (0.65-4.29)<br>0.1956	1.31 (0.51-3.39)<br>0.5784
t(4;14) IGH/FGFR3<br>Present<br>Absent<br>*No data: n=40*	17<br>25	1.42 (0.60-3.36)<br>0.3540	1.07 (0.45-2.53)<br>0.8731	22<br>45	2.62 (0.81-8.46)<br>0.0190*	3.22 (1.20-8.63)<br>0.0206*
t(11;14) IGH/CCND1<br>Present<br>Absent<br>*No data: n=30*	9<br>25	2.49 (0.85-7.35)<br>0.0119*	2.34 (0.99-5.51)<br>0.0537#	45<br>46	1.18 (0.39-3.61)<br>0.7491	0.55 (0.11-2.70)<br>0.4620
t(14;16) IGH/MAF<br>Present<br>Absent<br>*No data: n=30*	9<br>25	1.30 (0.14-12.23)<br>0.7924	1.56 (0.21-11.80)<br>0.6657	28<br>46	1.89 (0.13-28.23)<br>0.5172	6.64 (0.76-57.95)<br>0.0883
Other IGH rearrangement<br>Present<br>Absent	17<br>25	1.13 (0.53-2.38)<br>0.7374	1.06 (0.46-2.44)<br>0.8890	NR<br>45	1.19 (0.43-3.28)<br>0.7157	0.93 (0.32-2.71)<br>0.8994

No data: n=30						
amp 1q21/CKS1B<br>Present<br>Absent<br>*No data: n=105*	14<br>25	1.20 (0.38-3.73)<br>0.7509	0.73 (0.13-4.03)<br>0.7202	NR<br>NR	1.05 (0.18-6.21)<br>0.9605	N/a<br>0,9761
KIAA1524 genotype<br>(686G>A, rs2278911)<br>CC<br>CT or TT	27<br>12	0.40 (0.22-0.74)<br>0.0001*	0.53 (0.28-1.00)<br>0.0542*	46<br>24	0.41 (0.19-0.85)<br>0.0016*	0.55 (0.24-1.25)<br>0.1563
KIAA1524 genotype<br>(686G>A, rs2278911)<br>TT<br>CC or CT	6<br>25	4.67 (0.73-29.93)<br>0.0002*	7.57 (2.54-22.61)<br>0.0003*	8<br>45	4.39 (0.59-32.61)<br>0.0017*	6.89 (1.95-24.40)<br>0.0029*

Abbreviations: B2M – beta-2-microglobulin, CI – confidence interval, CRP – C-reactive protein, eGFR – estimated glomerular filtration rate, HR – hazard ratio, ISS - Multiple Myeloma International Staging System, LDH - lactate dehydrogenase, mOS – median overall survival, N/d – not determined, NR- not reached, *p* - statistical significance, WHO -World Health Organization. * - statistically significant result, # - trend toward significance,
